# Vancomycin-loaded silk fibroin microspheres in an injectable hydrogel for chronic osteomyelitis therapy

**DOI:** 10.3389/fbioe.2023.1163933

**Published:** 2023-06-01

**Authors:** Peng Zhang, Yusheng Sun, Haizhen Yang, Dong Liu, Feng Zhang, Yu Zhang, Wentao Zhong, Baoqi Zuo, Zhiqiang Zhou

**Affiliations:** ^1^ Department of Orthopedics, The Second Affiliated Hospital of Soochow University, Suzhou, China; ^2^ National Engineering Laboratory for Modern Silk, College of Textile and Clothing Engineering, Soochow University, Suzhou, China; ^3^ State Key Laboratory of Radiation Medicine and Protection, Soochow University, Suzhou, China; ^4^ Health Management Center, The Second Affiliated Hospital of Soochow University, Suzhou, China

**Keywords:** chronic osteomyelitis, silk fibroin, microspheres, vancomycin, hydrogel

## Abstract

**Introduction:** Chronic osteomyelitis remains a clinical challenge in orthopedics.

**Methods:** In this study, silk fibroin microspheres (SFMPs) loaded with vancomycin are entrapped in an injectable silk hydrogel to form a vancomycin delivery system for treatment of chronic osteomyelitis.

**Results and Discussion:** Vancomycin showed continuous release from the hydrogel for up to 25 days. The hydrogel shows strong antibacterial activity against both Escherichia coli and Staphylococcus aureus and a long antibacterial duration of 10 days without a decrease in the antibacterial effect. The injection of vancomycin-loaded silk fibroin microspheres entrapped in the hydrogel into the infected site of rat tibia reduced bone infection and improved bone regeneration compared with other treatment groups.

**Conclusion:** Thus, the composite SF hydrogel features a sustained-release profile and good biocompatibility, making it promising for application in osteomyelitis treatment.

## 1 Introduction

Chronic osteomyelitis is a progressive inflammatory process caused by bacterial infection (*Staphylococcus aureus*), characterized by bone destruction and sequestrum formation (LEW and WALDVOGEL, 2004). Its management remains challenging to clinical physicians, with a multidisciplinary approach involving radiologists, microbiologists, and orthopedic surgeons (NANDI et al., 2016). Currently, the combination of surgical debridement of the infected site and systemic antibiotic administration is considered to treat chronic osteomyelitis. However, this treatment regime could result in nonunion bone defect formation of the debrided site, which ultimately affects the bone healing process and may lead to amputation. Meanwhile, the Infectious Diseases Society of America, the American Society of Health-System Pharmacists, and the Society of Infectious Diseases Pharmacists published consensus guidelines on therapeutic monitoring of vancomycin in adults. After an exhaustive review of the literature, the guideline committee concluded that available evidence suggests that the ratio of the 24-h area under the concentration–time curve (AUC_0–24_) to the MIC (AUC/MIC ratio) drives the efficacy of vancomycin against infections with *Staphylococcus aureus* (RYBAK et al., 2009; NEELY et al., 2014)*.* However, systemic antibiotic administration requires a long term and large dose because localized bacterial plaque can inhibit antibiotic diffusion from the blood to the infection site, which may lead to various side effects, including drug resistance and nephrotoxicity. Additionally, it is difficult to achieve an effective local antibiotic concentration due to vascular injury in the infected bone. To overcome these challenges, a local drug delivery system has been established as an effective approach to avoid potential toxicity and provide a sustained high concentration of antibiotics at the infection site.

At present, many kinds of drug carriers, such as gels, bone cements, nano/microspheres, films, and porous scaffolds, have been designed and used to carry antibacterial agents for sustained release (COBB et al., 2020; FANG et al., 2021). Li et al. (2015) prepared mesoporous silica nanoparticles functionalized with aminopropyltriethoxysilane (MSN-APS) loaded with vancomycin (VAN) and then mixed with calcium sulfate bone cement. The so-called VAN@APS-MSN cement significantly improved the sustained-release time of VAN up to 10 days. Ahadi studied the preparation of a silk fibroin (SF)/oxidized pectin (OP) hydrogel with the electrospinning of an ammoniacal poly(L-lactic acid) (PLLA) fiber (AHADI et al., 2019). The effect of the PLLA fiber enhanced the drug sustained-release effect of antibacterial agents. Besheli loaded vancomycin into silk fibroin nanoparticles (VSFNPs) (BESHELI et al., 2017). VSFNPs were rich in silk fibroin scaffolds, and it was found that the sustained-release system had a longer release cycle at pH = 4.5. According to previous studies, nano/microspheres have a higher specific surface area, adjustable release kinetics, and sufficient stability than hydrogels and porous scaffolds, making them advantageous in the treatment of osteomyelitis (WANG et al., 2010; BESHELI et al., 2017).

Many natural compounds or synthetic polymer microspheres with sustained-release effects and degradation performance have been used in the treatment of osteomyelitis, including polylactic acid glycolic acid (PLGA), silica, chitosan, and gelatin (CEVHER et al., 2006; WANG et al., 2017; AKSOY et al., 2019; GONG et al., 2019). However, the problems of complicated preparation methods and rapid degradation of the microspheres have not been well-solved. As a natural polymer, mulberry silk is composed of silk fibroin and sericin. The outer layer of the silk fibroin fiber is wrapped with sericin, with the content of silk fibroin accounting for 75 % and that of sericin for 25 % approximately (KOH et al., 2015). Silk fibroin is the structural protein of the silk fiber, which is mainly composed of glycine, alanine, serine, and other amino acids. The structure of aggregated silk fibroin can be divided into an amorphous structure and a crystalline structure, namely, silk I and silk II, respectively; silk I has a metastable structure, and silk II has a *β*-sheet structure. Under certain conditions, metastable silk I can be transformed into stable silk II (KIM et al., 2004). Therefore, with its own composition and structural characteristics, silk fibroin as a biomaterial has the advantages of excellent biocompatibility, biodegradability, low immunogen reactivity, and plasticity.

In the clinical treatment of chronic osteomyelitis, the affected area often presents irregular morphology, and the use of microspheres as drug carriers for chronic osteomyelitis is often affected by its loose structure, unable to effectively fill the affected area and provide mechanical support. Therefore, based on the existing research studies, microspheres are often combined with hydrogels and other carriers to form a sustained-release system to expand its application scope. The injectable hydrogel can match the complex wound of chronic osteomyelitis with its advantages of filling and minimally invasive injection and will become a new choice of a drug treatment carrier for chronic osteomyelitis. Through a preliminary study in the laboratory, it was found that the CaCl_2_/formic acid (Ca–FA) dissolution system could decompose silk fibroin into a nanofiber and further prepare a hydrogel with the morphology of an extracellular matrix (ECM) fiber network to simulate the ECM environment (MING et al., 2014). SUN et al. (2022) dissolved silk fibroin in the Ca/FA dissolution system and obtained an aqueous silk fibroin nanofiber (SF-NF) solution. An injectable SF–NF hydrogel with a faster gel rate and longer vancomycin sustained-release period was further prepared.

In this study, silk fibroin microspheres (SFMPs) were used as drug carriers to load vancomycin (VANCO) to form drug microspheres (VSFMPs), and then a silk fibroin nanofiber (SF–NF) solution was used to prepare an injectable hydrogel as a carrier for VSFMPs (SF-NF@VSFMPs), which forms a modified delivery system for chronic osteomyelitis. SF-NF was obtained by sequentially dissolving silk fibroin in two solutions: CaCl_2_/FA and CaCl_2_/C_2_H_5_OH/H_2_O (SUN et al., 2022). The injectable hydrogel formed by blending the SF-NF with low-molecular weight polyethylene glycol (PEG400) has good injectable properties and stability (WANG et al., 2015). In addition, the release of VANCO showed a pH response and a more ideal release curve in the sustained-release system of VSFMPs and SF-NF@VSFMPs. Our research results provide a new option in tissue engineering for future treatment of osteomyelitis.

## 2 Materials and methods

### 2.1 Materials

Raw silk was purchased from Haian, Jiangsu. Anhydrous sodium carbonate was provided by Qiangsheng Functional Chemical Co., Ltd. (Jiangsu, China). Anhydrous calcium chloride was provided by China National Pharmaceutical Group Chemical Reagent Co., Ltd. (Shanghai, China). Anhydrous ethanol and formic acid (98%) were purchased from Lingfeng Chemical Reagent Co., Ltd. (Shanghai, China). PEG 400, PEG 6000, and Tween-20 were provided by Suzhou Shengnuokang Biotechnology Co., Ltd (Suzhou, China). VANCO was supplied by Aladdin Biochemical Technology Co., Ltd. (Shanghai, China). Dulbecco’s modified Eagle’s medium (DMEM, 4.5 g L^−1^ D-glucose) was purchased from Gibco, United States. Fetal bovine serum (FBS) was produced by Procell (Wuhan, China). Streptomycin sulfate, ampicillin, 0.25% trypsin, dimethyl sulfoxide (DMSO), paraformaldehyde and Triton X-100, hematoxylin and eosin (H&E), and Masson’s trichrome stain (Masson) were purchased from Sigma (United States). CCK-8, fluorescein isothiocyanate (FITC), and Hoechst 33258 were provided by Beyotime Biotechnology Inc. The water used in this laboratory was homemade deionized water.

### 2.2 Preparation of silk fibroin (SF) and SF–NF

To remove sericin from the surface of raw silk, the raw silk was first degummed in 0.05 wt% Na_2_CO_3_ solution with a bath ratio of 1:40 for 30 min, and then the remaining Na_2_CO_3_ and sericin were washed with deionized water. This step was repeated three times to obtain degummed silk. The silk was removed and dried at 60°C to obtain degummed silk.

#### 2.2.1 Preparation of SF

A total of 40 mL 9.3 M LiBr solution was added to 10 g degummed silk, sealed, and dissolved at 60°C for 4 h after the silk was completely wetted. The dissolved degummed wire was transferred into a dialysis bag (8,000–14,000 Da), and dialysis was carried out in deionized water for 3 days to obtain SF.

#### 2.2.2 Preparation of the SF–NF

SF was dissolved in Ca/FA to form an SF membrane containing nanofibers (ZHANG et al., 2015). Because the crystal structure of the SF fiber was destroyed by Ca/FA, CaCl_2_/ethanol/H_2_O could dissolve the regenerated silk fibroin membrane at room temperature with a molar ratio of 1:2:8 for 4 h, in which the mass ratio of the silk fibroin membrane to CaCl_2_/ethanol/H_2_O was 1:20. After 72 h of dialysis (8,000–14,000 Da dialysis bag), the SF–NF was obtained. After dialysis, the SF and SF–NF were removed and concentrated at 60°C, and the concentration was tested. Finally, it was diluted for further use.

### 2.3 Preparation and characterization of VSFMPs

#### 2.3.1 Preparation of SFMPs

SFMPs were prepared by the self-assembly method. About 20 % w/v PEG 6000 and Tween-20 were blended with 3 wt% SF at volume ratios of 1:4 and 1:2, respectively, stirred for 10 min, and then stored at −20°C for 24 h. A centrifuge (KaiT, TGL20M, China) was used to centrifuge the thawed sample at 12, 000 r min^−1^ for 15 min to remove the supernatant and resuspend the precipitate with deionized water. This step was repeated three times before freeze-drying to obtain SFMPs.

#### 2.3.2 Preparation of VSFMPs

An amount of 50 mg of SFMPs was added to 10 mL VANCO solution with different concentrations (0.1, 0.3, 0.5, and 0.7 mg mL^−1^), denoted as 0.1 VSFMPs, 0.3 VSFMPs, 0.5 VSFMPs, and 0.7 VSFMPs, respectively. The mixture was centrifuged 24 h later at 2,000 r min^−1^ for 10 min to collect VSFMPs. The content of VANCO in the sample was determined by UV spectrophotometry (SPECORD S600, Germany) at 280 nm.

#### 2.3.3 SEM

SFMPs and VSFMPs were bound to the sample table with conductive adhesive, and gold spraying was performed for 90 s. The samples were observed by scanning electron microscopy (SEM, Regulus 8100, Hitachi) at an accelerated voltage of 3.0 kV, and ImageJ was used for SFMP and VSFMP size analysis after SEM characterization.

#### 2.3.4 ZETA potential

To characterize the changes in the ZETA potential for the surface of VSFMPs (Malvern Zetasizer Nano ZS90, England) after VANCO loading, the ZETA potential of the SF and VANCO was first measured, and then the surface ZETA potential of SFMPs was measured by dispersing them in deionized water. Finally, VSFMPs were dispersed in deionized water to measure their surface ZETA potential.

#### 2.3.5 FT-IR

Fourier transform infrared (FT-IR) spectra of VANCO, SFMPs, and VSFMPs in the wave number range of 400–4,000 cm^−1^ were recorded using an attenuated total reflection spectrometer (Nicolet iS5). The number of scans was 32, and the resolution was 4 cm^−1^.

#### 2.3.6 XRD

The crystal structures of VANCO; SFMPs; and 0.1, 0.3, 0.5, and 0.7 VSFMPs were analyzed by X-ray diffraction (XRD, D8 Advance, Germany) with scanning angles of 5°–60° and a step size of 0.02°.

#### 2.3.7 Drug release

PBS solutions with different pH values (4.5 and 7.4) were used for sustained release. VSFMPs were dispersed in 10 mL sustained-release solution at room temperature. Every 24 h, 2 mL of the PBS sustained-release supernatant was replaced with the same amount of fresh PBS, and the sustained-release supernatant was analyzed by UV spectrophotometry (SPECORD S600, Germany) at 280 nm to calculate the drug content in the supernatant.

### 2.4 Performance characterization of SF-NF@VSFMPs

#### 2.4.1 Drug release of SF-NF@VSFMPs

##### 2.4.1.1 Preparation of the SF-NF@VSFMP injectable hydrogel

Injectable hydrogels were prepared by mixing PEG 400 and SF–NF (WANG et al., 2015). After a certain amount of the VSFMPs and SF–NF was blended evenly, the SF-NF@VSFMP-injectable hydrogel loaded with VSFMPs was formed at an SF–NF and PEG 400 volume ratio of 1:1.

##### 2.4.1.2 Drug release

To evaluate the release of VANCO in the SF-NF@VSFMP injectable hydrogel, a certain amount of VSFMPs was added to 5% SF–NF, blended with PEG 400 at a volume ratio of 1:1 to form hydrogels, and then placed in 10 mL PBS sustained-release solution with pH = 4.5 or pH = 7.4. Every 24 h, 2 mL PBS sustained-release supernatant was replaced with the same amount of fresh PBS, and the drug content of the sustained-release supernatant was determined by UV spectrophotometry (SPECORD S600, Germany) at 280 nm.

#### 2.4.2 Antibacterial performance

In the biocompatibility and antibacterial performance experiments, to prevent VANCO from being inactivated under high temperature and high pressure, the SF-NF@VSFMPs were sterilized under ultraviolet light for 24 h.

To evaluate the inhibitory effect of SF-NF@VSFMP injectable hydrogel on *Staphylococcus aureus* (ATCC 6538) and *Escherichia coli* (ATCC 11229), VSFMPs were added to 5% SF–NF and blended with PEG 400 at a volume ratio of 1:1. Then, 20 mL nutrient broth with a concentration of 18 g L^−1^ was added into a conical flask and then sterilized at 121°C for 30 min. After the broth cooled down, a sterilized harvesting ring was used to pick out *Staphylococcus aureus* and *Escherichia coli* colonies and transfer them to the broth for 18–20 h.

##### 2.4.2.1 Drug dissolution test of SF-NF@VSFMP injectable hydrogel (agar diffusion method)

The nutrient broth and PBS were used to dilute *Staphylococcus aureus* and *Escherichia coli* to the viable bacteria cell count of 1 × 10^5^ CFU mL^−1^. An amount of 20 mL of agar was poured into 9-cm bacteriological Petri dishes. Subsequently, 100 μL of broth containing *Staphylococcus aureus* and *Escherichia coli* (1 × 10^5^ CFU mL^−1^) was dispersed on a solidified agar medium. The SF-NF@VSFMP injectable hydrogel was then carefully injected into the agar surface containing *Staphylococcus aureus* and *Escherichia coli*, respectively. After 24 h of incubation at 37°C, the inhibition zone around each sample was observed, and its width was measured. At least three sets of parallel samples for each sample and at least three points for each sample were measured. The width of the inhibition zone is calculated by the following formula: width of the inhibition zone = (outer diameter-inner diameter)/2.

##### 2.4.2.2 Antimicrobial activity of the SF-NF@VSFMP injectable hydrogel.

First, *Staphylococcus aureus* and *Escherichia coli* were diluted with the nutrient broth and PBS to the viable bacteria cell count of 3 × 10^5^ CFU mL^−1^, 5 mL of the diluted bacterial solution was added to the conical flask, and 1 mL of SF-NF@VSFMP injectable hydrogel was added into the conical flask, exposed to the flask for 18 h by shaking.

Second, three sample bottles named A, B, and C were prepared; 1 mL of the aforementioned bacterial solution was added to sample bottle A equipped with 9 mL PBS to dilute 10 times; 1 mL of the bacterial solution from sample bottle A was added to sample bottle B equipped with 9 mL PBS to dilute 100 times; 1 mL of the bacterial solution from sample bottle B was added to sample bottle C equipped with 9 mL PBS to dilute 1,000 times.

Finally, 1 mL of bacterial solution from dilution bottles A, B, and C was taken and evenly mixed with nutrient agar. After 24 h of incubation at 37°C, the colonies were counted, and the bacteriostatic rate (W) of the samples was obtained by the following formula:

$$W=\left(M-N\right)/M\times 100\%.$$

M is the mean colony count of control samples, and N is the mean colony count of samples.

#### 2.4.3 *In vitro* biocompatibility characterization

A mouse embryonic osteoblast line (MC3T3) was selected to evaluate the biosafety of the prepared SF-NF@VSFMPs. MC3T3 cells were cultured in Dulbecco’s modified Eagle’s medium (DMEM, 4.5 g L^−1^ D-glucose) containing a 1% (V/V) mixture of penicillin and streptomycin and 10% (V/V) fetal bovine serum (FBS). When MC3T3 cells entered the third to fifth generation, 80%–90% of the cells were removed via digestion with 1 mL trypsin for approximately 30 s; 1 mL of fresh DMEM containing FBS and antibiotics was added to stop digestion. The digested cell pellet was centrifuged at 900 r min^−1^ for 5 min. The cell density was adjusted so that each sample was inoculated with 10 μL of cell suspension, and the final inoculated cell density was 1 × 10^4^ cells well^−1^. The samples (0.1 VSFMPs, 0.3 VSFMPs, 0.5 VSFMPs, and 0.7 VSFMPs) were placed in a 37°C cell culture box with 5% CO_2_ and incubated for 2 h. Then, fresh DMEM containing FBS and antibiotics was added. The medium was replaced every 1–2 days, and cell proliferation and growth were observed at the corresponding time points.

##### 2.4.3.1 Qualitative assay of cell proliferation by laser confocal microscopy

After 1, 4, and 7 days of cell inoculation, the cell culture medium was removed, and the cells were gently washed three times with high-temperature sterilized PBS in preparation for staining. First, the cells were fixed with 500 μL 4% paraformaldehyde (PFA) solution for 30 min. Second, 0.01% Triton X-100 solution was prepared at 37°C, and 500 μL was added for 10 min. Finally, FITC and Hoechst 33258 solution were prepared at a ratio of 1:1,000. Then, 500 μL FITC solution was added, and the samples were incubated for 1 h. The FITC solution was then removed, the samples were washed with PBS three times, and finally, 500 μL Hoechst 33258 solution was added and incubated for 10 min. The material was observed under a confocal laser microscope (Zeiss LSM 800, Germany), where the cytoskeleton was stained green and the nucleus was stained blue.

##### 2.4.3.2 CCK-8 kit was used to quantitatively assay cell proliferation

After 1, 4, and 7 days of culture, CCK-8 and the medium were prepared at a ratio of 1:10 in the dark, and an appropriate amount of the solution was added to each well. The cells were incubated in a cell culture box in the dark for 2 h, and then 200 μL of the supernatant was added to a 96-well plate via a micropipette. The OD was measured at 450 nm using a microplate reader (H2 Microplate Reader, BioTech, United States). All experimental samples were set as six parallel samples.

#### 2.4.4 *In vivo* biocompatibility

Based on the results of *in vitro* compatibility and antimicrobial activity, SF-NF@VSFMP injectable hydrogel-loaded 0.7 VSFMPs were selected for *in vivo* studies. Meanwhile, the injectable SF–NF hydrogel, which was not loaded with VSFMPs, was used as a control.

Male SD rats (4 weeks old, 160–180 g, Zhaoyan New Drug Research Center Co., Ltd., Suzhou, China) were used to assess the *in vivo* biocompatibility of the SF-NF@VSFMP injectable hydrogel. All animal procedures were conducted in accordance with the National Institutes of Health Guide for Care and Use of Laboratory Animals and approved by the Institutional Animal Care and Use Committee of Soochow University. The animals were anesthetized with trichloroacetaldehyde hydrate (50 mg 100 g^−1^) before surgery. A 1.5 cm longitudinal incision was made at the proximal end of the right tibia to separate the surrounding skin tissue and expose the tibial plateau. A puncture needle was used in the middle of the tibial plateau to open a hole, and a 2-mm Kirschner needle was used to enlarge the hole and expose the bone marrow cavity. The experimental animals were divided into two groups. The control group was only injected with 50 μL of a 1 × 10^7^ CFU mL^−1^ *Staphylococcus aureus* suspension and 100 μL injectable SF–NF hydrogel. The experimental group was injected with 50 μL of 1 × 10^7^ CFU mL^−1^ *Staphylococcus aureus* suspension, followed by 100 μL of the SF-NF@VSFMP injectable hydrogel. The bone wax was used to close the hole, and the wound was sutured after washing three times with saline. Antibiotics were not used during and after the operation. Then, 1 week after the operation, the rats were anesthetized again on the operating table, and the original incision was dissected to re-expose the drilling holes of the tibial plateau. Debridement was performed on the affected limbs and rinsed with saline three times. Then, steel brushes and dental burs were used to eliminate necrotic and infected tissues repeatedly and rinsed three times with saline again. The blank control group (a) was implanted with an injectable SF–NF hydrogel (n = 5); the experimental group (b) was implanted with an SF-NF@SFMPs injectable hydrogel (n = 5). The wound was cleaned, and the incision was closed. Routine feeding was carried out after the operation, and 3 weeks after the operation, the rats were euthanized, and bone specimens were collected.

Three weeks after the operation, the micro-CT imaging of the affected limbs was performed. After the integrated tibia was dissected and weighed, the specific infected bone tissues of the upper one-third segment of the tibia were stained with H&E and Masson’s trichome and observed under a microscope.

### 2.5 Statistical analysis

All quantitative data were expressed as the means + s.d. All the statistical analyses were performed by SPSS 16.0. A value of *p* < 0.05 was considered significant. *p < 0.05, **p < 0.01, and ***p < 0.001.

## 3 Results and discussion

This work aimed to study a modified treatment for chronic osteomyelitis. Chronic osteomyelitis is usually caused by bone avascular necrosis and formation of dead bone caused by *Staphylococcus aureus* infection in the bone marrow cavity. Conventional treatment consists of systemic antibiotic treatment for 4–6 weeks after surgical debridement, but this treatment usually causes significant damage to the patient’s body and side effects caused by high concentrations of drug treatment cause side effects. Therefore, this paper studies a silk fibroin microsphere drug delivery carrier (VSFMPs) with long-term sustained release of VANCO to treat chronic osteomyelitis. To further reduce the negative impact on patients, injectable hydrogels equipped with VSFMPs to form a SF-NF@VSFMP injectable hydrogel are used, which can prolong the sustained-release cycle of drugs and achieve a minimally invasive treatment effect.

### 3.1 Preparation of SF–NF, VSFMPs, and SF-NF@VSFMP injectable hydrogel

**FIGURE 1:**
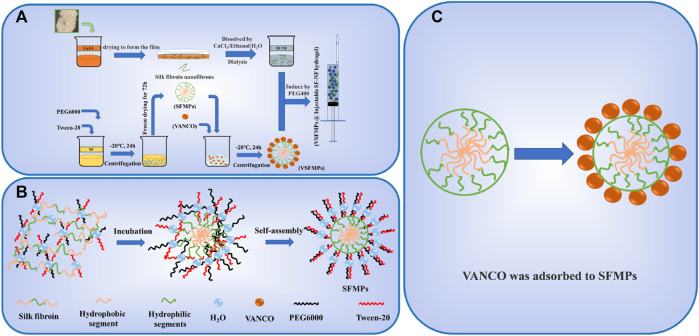
Schematic illustration of the fabrication and interaction of SF-NF@VSFMPs. **(A)** Preparation of SFMPs, VSFMPs, and SF-NF@VSFMPs. **(B)** Schematic illustration of the formation of SFMPs. **(C)** Physical adsorption between SFMPs and VANCO.

Figure 1A shows the preparation process of SF–NF, VSFMPs, and SF-NF@VSFMP injectable hydrogel. The SF–NF was prepared by two-step dissolution in this study. When the hydrophobic amino acid was dominant, the phase separation first occurred when the SF and hydrophilic PEG were blended, and the SF would form dispersed phase droplets in the continuous-phase PEG solution. Since the PEG was a polar solvent and Tween-20 could reduce the liquid surface tension, with the ongoing incubation, the dispersed phase of free water and bound water molecules on the surface of the SF would be transferred more to the continuous phase, and SF would lose water molecules and expose a hydrophobic region. Then, the SF gradually gathered under the hydrophobic forces and precipitated to form stable SF microspheres (Figure 1B) (WU et al., 2016). VSFMPs were obtained by freeze-drying after mixing SFMPs with VANCO solutions of different concentrations (0.1, 0.3, 0.5, and 0.7 mg mL^−1^) for 24 h at room temperature (WU et al., 2016). To prepare the SF-NF@VSFMP injectable hydrogel, a certain amount of VSFMPs was added to 5% SF–NF and blended with 85% PEG 400 at a volume ratio of 1:1 at room temperature (WANG et al., 2015).

Similar to the methods observed in previous research, degummed silk was dissolved in a CaCl_2_/FA dissolution system, air-dried, and then dissolved into a silk fibroin membrane with a CaCl_2_/ethanol/H_2_O ternary dissolution system to obtain the SF–NF. It was found that the crystal structure of the SF–NF is mainly a typical transition state silk I structure, which easily changes into a stable silk II structure under external stimulation and induction, thus giving the SF–NF the ability to quickly gel and be injected (LU et al., 2010; SUN et al., 2022).

### 3.2 Characterization of VSFMPs

**FIGURE 2:**
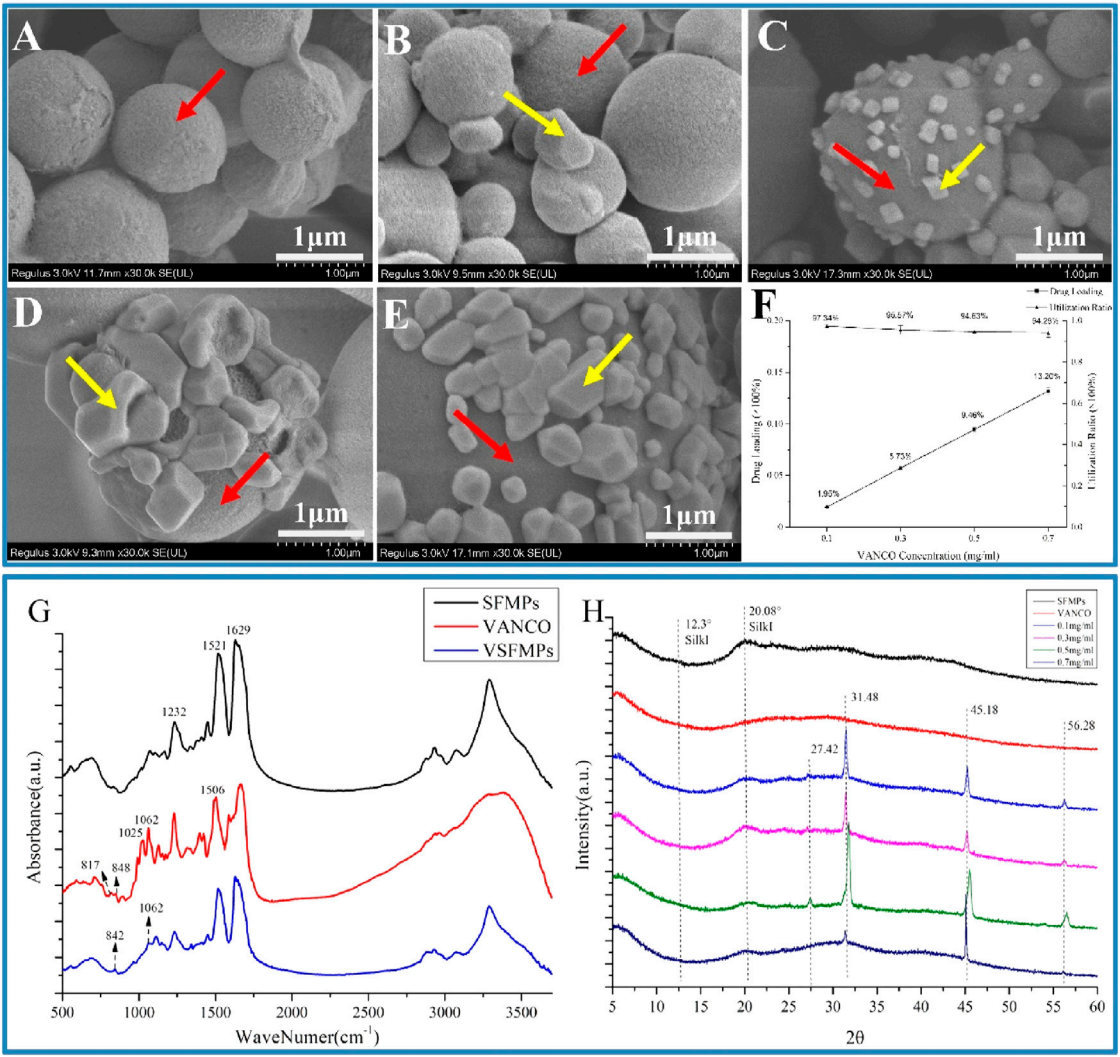
Morphologies and structural analyses of the SF-NF@VSFMP sustained-release system. **(A)** SFMPs; **(B)** 0.1 VSFMPs; **(C)** 0.3 VSFMPs; **(D)** 0.5 VSFMPs; **(E)** 0.7 VSFMPs; **(F)** drug loading ratio and utilization ratio of VSFMPs; **(G)** FT-IR measurement of SFMPs, VANCO, and VSFMPs; **(H)** XRD measurement of SFMPs, VANCO, and VSFMPs, where the red arrow is the surface of SFMPs and the yellow arrow is the surface of VANCO, where 0.1, 0.3, 0.5, and 0.7 mg mL^−1^ represent 0.1, 0.3, 0.5, and 0.7 VSFMPs, respectively.

As shown in Figure1C and Figure 2A, when the SFMP surface was loaded with VANCO, SFMPs with smooth surfaces clearly show VANCO attachment. As shown in Figures 2B–E, VANCO was successfully loaded to SFMPs and could be observed on the surface of SFMPs. The drug loading rate and utilization rate of VANCO in VSFMPs are shown in Figure 2F, which shows that the drug loading rate of VANCO increases with its increasing concentrations. When the VANCO concentration was 0.1 mg mL^−1^, the drug loading rate of 0.1 VSFMPs was only 1.95%, but the drug utilization rate was the highest (97.34%). When the concentration of VANCO increased to 0.7 mg mL^−1^, the drug loading rate of 0.7 VSFMPs reached 13.20%, indicating that increasing the concentration of VANCO can significantly improve the drug loading rate.

The surface ZETA potential, structural characteristics, and sustained-release effect of VSFMPs were further analyzed. First, the size and ZETA potential of SFMPs were characterized. As shown in Table 1, the ZETA potential of SF and VANCO showed negative values of −4.08 and −10.00, respectively. In the process of self-assembly, negatively charged silk fibroin molecules gather together into balls, resulting in the SFMP surface ZETA potential being reduced to −22.17 mV. When VANCO is loaded on SFMPs, the ZETA potential of the SFMP surface decreased due to the physical adsorption of negatively charged VANCO (−9.98 mV). Meanwhile, with increasing VANCO concentration, the particle size of VSFMPs increases, all of which are greater than 1 μm. The reason for this phenomenon may be that the successful loading of VANCO results in its presence on the surface of SFMPs. In comparison, YANG et al. (2011) deposited a vancomycin/chitosan composite coating on the surface of the carrier by electrochemical deposition for the treatment of chronic osteomyelitis. KETONIS et al. (2009) supported using VANCO for the treatment of chronic osteomyelitis by etching the passivated carrier surface with H_2_SO_4_/H_2_O_2_ to generate an oxide layer. All the aforementioned methods have the disadvantage of being a complex preparation process. In this study, VANCO was loaded on SFMPs by means of physical adsorption, and the preparation process was simple and efficient.

**TABLE 1:** Size and ZETA potential distribution of SFMPs and VSFMPs.

Sample	Size (μm)	Zeta potential (mV)
SF		−4.12 ± 0.32
VANCO		−10.01 ± 0.21
SFMPs	0.93 ± 0.37	−22.17 ± 0.54
0.1 VSFMPs	1.13 ± 0.35	−39.10 ± 0.64
0.3 VSFMPs	1.09 ± 0.45	−34.70 ± 1.02
0.5 VSFMPs	1.01 ± 0.34	−36.47 ± 0.62
0.7 VSFMPs	1.05 ± 0.37	−38.87 ± 0.42

Fourier transform infrared spectroscopy (FT-IR) and X-ray diffraction (XRD) revealed the relationship between SFMPs and VANCO. The present study showed that the SF at 1,650–1,660 cm^−1^ (amide Ⅰ), 1,535–1,545 cm^−1^ (amide Ⅱ), and 1,235 cm^−1^ (amide Ⅲ) was a random coil, and that at 1,620–1,640 cm^−1^ (amide Ⅰ), 1,515–1,525 cm^−1^ (amide Ⅱ), and 1,265 cm^−1^ (amide Ⅲ) was a *β*-sheet structure (CHEN et al., 2001; HU et al., 2010). Figure 2G shows that SFMPs have three characteristic absorption peaks at 1,232, 1,521, and 1,629 cm^−1^, representing silk fibroin amide I (C=O stretching vibration and N-H bending vibration), amide II (N-H bending and C-N stretching vibration), and amide III (N-H bending and C-N stretching vibration), respectively. VANCO also has characteristic absorption peaks at 1,062, 1,025 (stretching vibration of C-N and C-O, respectively), 817, and 848 cm^−1^ (BESHELI et al., 2018; AHADI et al., 2019). At the same time, it can be observed that VSFMPs at 842 and 1,062 cm^−1^ have a new characteristic absorption peak that is not shown in the SFMP infrared spectrum, confirming the existence of VANCO in SFMPs.

The XRD analysis (Figure 2H) shows that SFMPs have characteristic peaks at 12.3° and 20.08°, which are typical of silk I structure (LU et al., 2010). The results showed that the secondary structure of SILK I was formed in the process of self-assembly. At the same time, the characteristic absorption peaks of 0.1, 0.3, 0.5, and 0.7 VSFMPs exhibited characteristic absorption peaks at 27.42°, 31.48°, 45.18°, and 56.28°, respectively. This shows that VANCO was successfully loaded and had good compatibility. However, it is worth noting that after VANCO was loaded on SFMPs, the characteristic peak of silk I in SFMPs disappeared at 12.3°, which may be because the secondary structure of silk fibroin was changed in the process of VANCO aggregation crystallization. The VANCO crystallization peak appears at a diffraction angle corresponding to that of VSFMPs, indicating that VANCO exists in VSFMPs in crystal form.

### 3.3 Drug release of VSFMPs and SF-NF@VSFMPs

**FIGURE 3:**
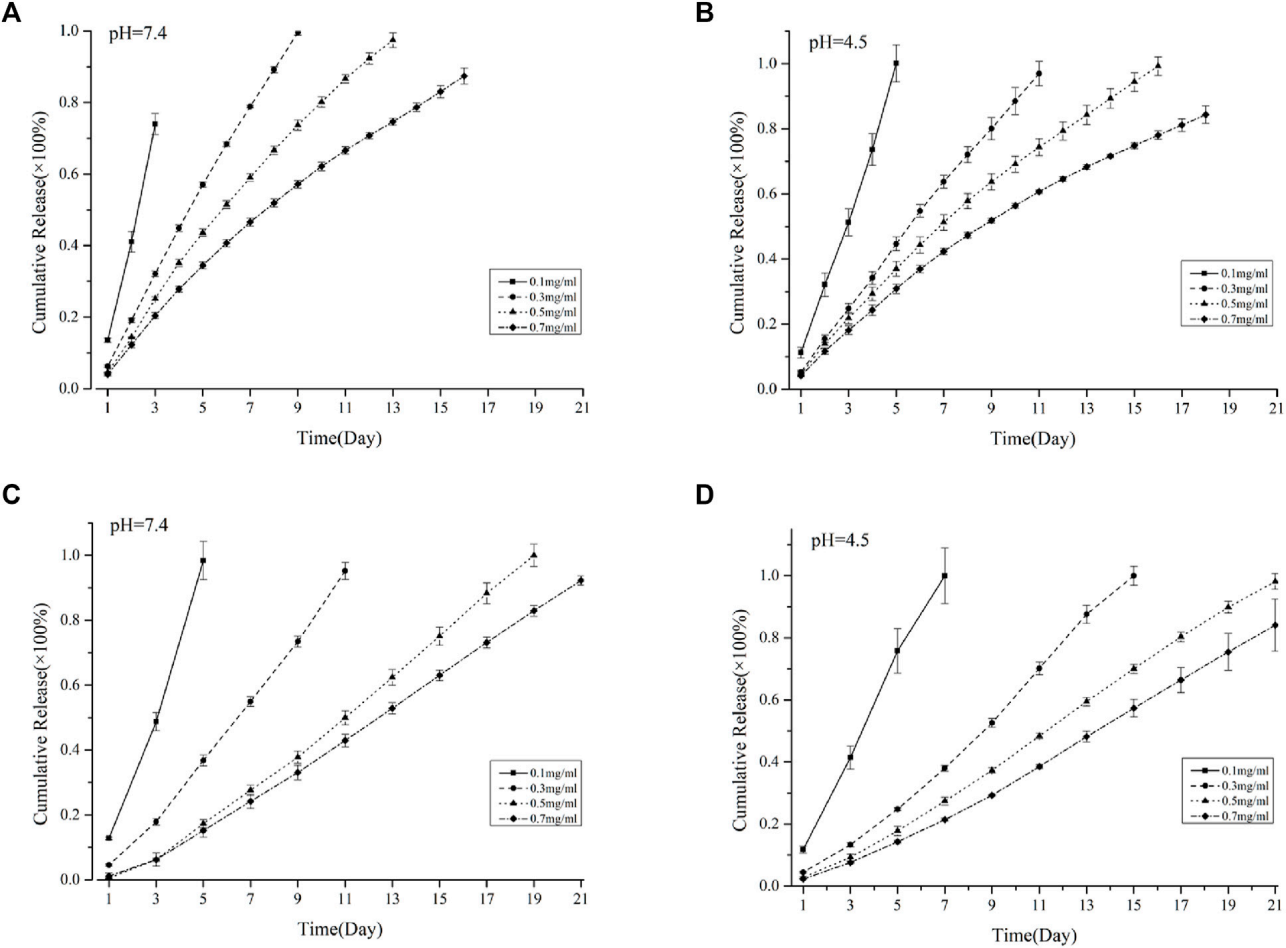
Cumulative release of VANCO from VSFMPs at **(A)** pH = 7.4 and **(B)** pH = 4.5. Cumulative release of VANCO from the SF-NF@VSFMP sustained-release system at **(C)** pH = 7.4 and **(D)** pH = 4.5, where 0.1, 0.3, 0.5, and 0.7 mg/mL represent the SF-NF@VSFMP sustained-release system loaded with 0.1, 0.3, 0.5, and 0.7 VSFMPs, respectively.

According to the literature reports, VANCO has different sustained-release effects in different pH environments, and osteomyelitis is affected by the acidic environment (LEW and WALDVOGEL, 2004; BESHELI et al., 2017).Therefore, to explore the sustained release of VANCO in different environments, VANCO sustained release was carried out under the conditions of pH 4.5 and 7.4. Figures 3A, B shows the sustained release of the VSFMP under two pH environments. Among them, 0.1 VSFMPs clearly showed a rapid initial release in both pH environments, and VANCO was completely released within 4 days. However, 0.3, 0.5, and 0.7 VSFMPs released the drug at a slow rate, without a clear rapid release at the start. The longest sustained release was 18 days at pH = 4.5, indicating that the release of VANCO from VSFMPs had a certain pH response. It could obtain a more long-term sustained release in an acidic environment.

The difference in the drug release phenomenon was because in the case of a low VANCO concentration, the VANCO drug-loading rate is low, and there is less association between VANCO and SFMPs, mainly in the form of weak ionic bonds, such as hydrogen bonds. When the concentration of VANCO increased, more drug molecules are bound to SFMPs through physical adsorption. Therefore, with the increase in the drug-loading rate, the drug release tended to be more constant. Meanwhile, the pH environments also affected the sustained-release rate. These results showed the ability of the VANCO to resist dissolution at low pH; thus, VSFMPs have a longer sustained-release time at pH of 4.5. Azagury et al. presented a quick fabrication method for preparing double-walled polymeric nanospheres (DW NPs). Insulin from the DW NPs showed zero release for the first 4 h at pH 3 (where insulin is very soluble) and continued to release for over 2 months. The aforementioned results indicated that the pH environment has an important impact on drug release.

Compared with nanospheres, silk fibroin microspheres have a longer sustained-release rate, which may be related to the size of SFMPs. Some research results showed that microspheres have a larger drug diffusion distance than nanospheres, which slows down the drug release rate and achieves a long-term sustained-release effect (WANG et al., 2020).

Because the VSFMPs were prepared by freeze-drying and added into the SF-NF injectable hydrogel, which was prepared by mixing PEG 400 and SF-NF, the process did not involve drug loss. The drug loading was exactly as designed. Figures 3C, D shows the sustained release of SF-NF@VSFMPs at pH 7.4 and 4.5. Among them, the SF-NF@VSFMPs loaded with 0.1 VSFMPs showed a dramatic initial release in the two pH environments at the initial stage. Compared with the 0.1 VSFMPs, the SF-NF@VSFMPs can sustain for a longer time, up to 7 days, at pH = 4.5. The results showed that VANCO in SF-NF@VSFMPs also had a certain pH response and could obtain a longer sustained release in an acidic environment. The sustained-release cycle of the SF-NF@VSFMPs is significantly better than that of VSFMPs, because in the process of sustained release, drugs are first released from VSFMPs into the injectable hydrogel and then from the injectable hydrogel to the sustained-release solution, which significantly improves drug diffusion and release distance, thus improving the sustained-release cycle of drugs. Besheli et al. loaded VANCO onto silk fibroin nanoparticles and then combined them with a silk fibroin scaffold for the treatment of chronic osteomyelitis (BESHELI et al., 2017). The sustained-release results showed that drug-loaded nanoparticles had a longer sustained-release cycle in the macro-sustained-release system, which could be increased by 16 days. ZHOU et al. (2018) loaded VANCO onto polylactic acid glycolic acid (PLGA) microspheres combined with polycaprolactone (PCL) scaffolds and found that the VANCO sustained-release system could release VANCO for 28 days. These results indicate that the combination of silk fibroin microspheres and injectable hydrogels can effectively prolong the sustained-release cycle of drugs. Moreover, hydrogel injection into areas of irregular chronic osteomyelitis is minimally invasive, which has broad research prospects in the treatment of chronic osteomyelitis in the future.

### 3.4 Antibacterial sensitivity of SF-NF@VSFMPs

**FIGURE 4:**
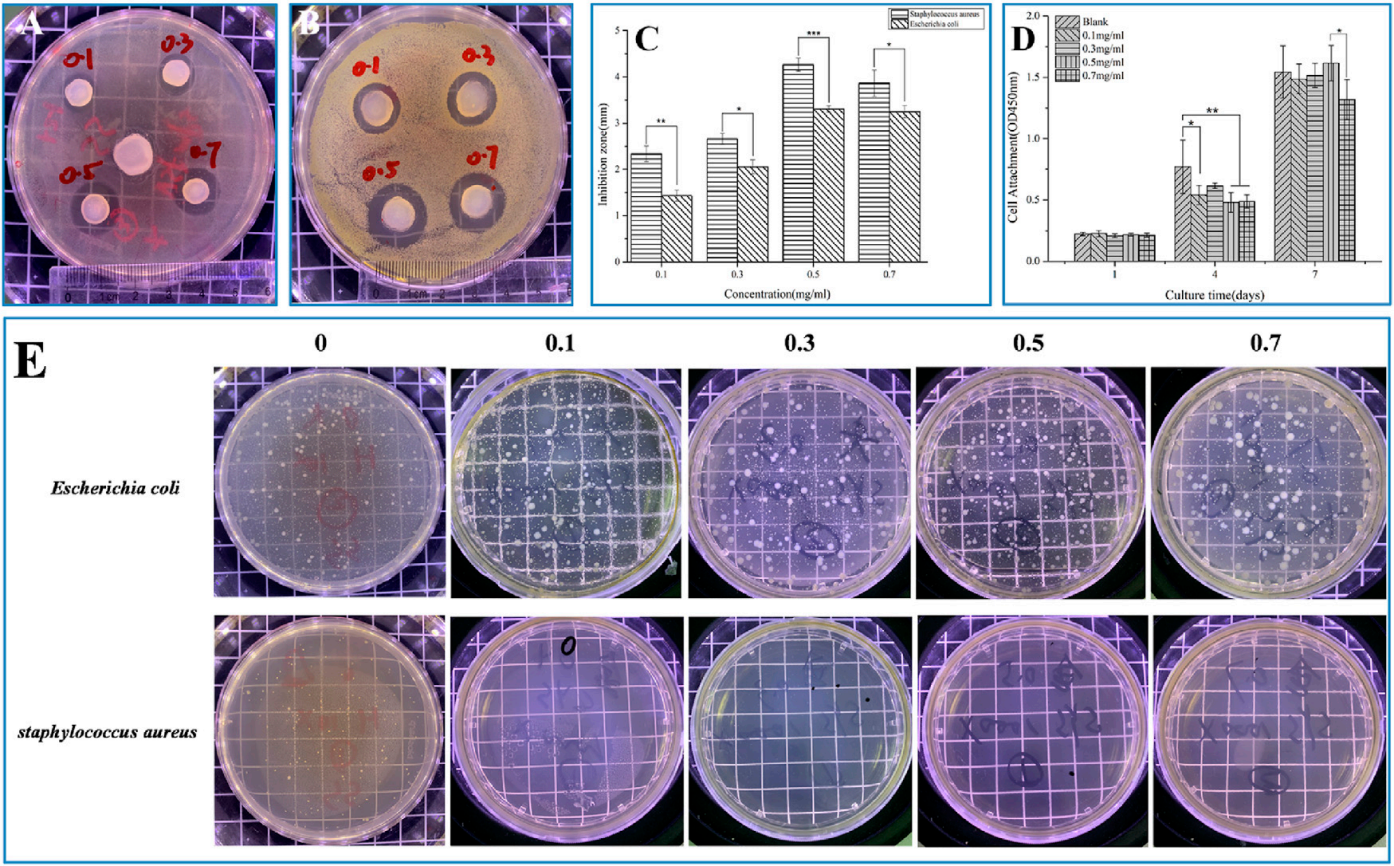
Antibacterial properties and biocompatibility of SF-NF@VSFMPs. Pictures of the agar diffusion test results of SF-NF@VSFMPs against **(A)** *Escherichia coli* and **(B)** *Staphylococcus aureus*; **(C)** diameter of the inhibition zone; **(D)** CCK-8 test results of cultured MC3T3 for 1, 4, and 7 days; and **(E)** Pictures of the oscillatory method results of SF-NF@VSFMPs against *Escherichia coli* and *Staphylococcus aureus,* where 0.1, 0.3, 0.5, and 0.7 mg mL^−1^ represent the SF-NF@VSFMP sustained-release system loaded with 0.1, 0.3, 0.5, and 0.7 VSFMPs, respectively. Note: *p < 0.05, **p < 0.01, and ***p < 0.001.

The main pathogen of chronic osteomyelitis is *Staphylococcus aureus*, and VANCO, as a glycopeptide antibiotic, is mainly used to treat Gram-positive bacterial infections, killing bacteria by changing the bacterial cell wall structure and metabolic mode (GARDETE and TOMASZ, 2014). Figures 4A, B show the bacterial sensitivity of SF-NF@VSFMPs in releasing VANCO, and the size of the negative control (excluding VSFMPs) inhibition zone was 0 mm (data not shown). Figure 4C shows the changes in the antibacterial zone of the SF-NF@VSFMPs to *Staphylococcus aureus* and *Escherichia coli*. The inhibitory effect of the system on *Staphylococcus aureus* was significantly better than that on *Escherichia coli*. For *Staphylococcus aureus*, the average diameter of the inhibition zone increased with increasing VANCO concentration. When treated with 0.5 VSFMPs, the average diameter of the inhibition zone reached the maximum, which was similar to that when treating with 0.7 VSFMPs. Therefore, the drug delivery system has a good inhibitory effect on *Staphylococcus aureus*.

As shown in Figure 4E, for *Staphylococcus aureus*, the number of colonies in the SF-NF@VSFMP medium was much smaller than that in the blank control medium. The bacteriostatic rates of SF-NF@VSFMPs with 0.1, 0.3, 0.5, and 0.7 VSFMPs to *Escherichia coli* were 80.71%, 78.81%, 77.16%, and 82.49%, respectively, while those of *Staphylococcus aureus* were 99.99%, 98.90%, 99.45%, and 99.99%, respectively, indicating that VANCO released from the sustained-release system has a significant inhibitory effect on *Staphylococcus aureus*.

### 3.5 *In vitro* biocompatibility characterization of SF-NF@VSFMPs

**FIGURE 5:**
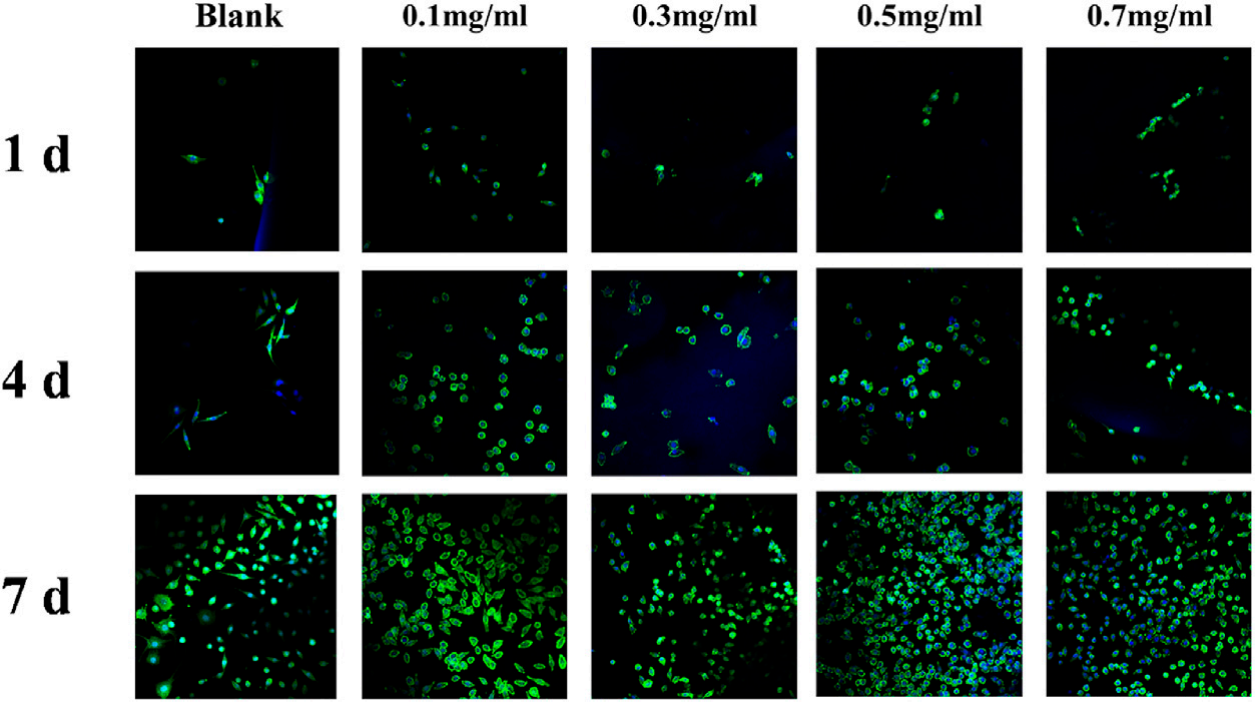
Laser confocal images of MC3T3 cultured in SF-NF@VSFMPs for 1, 4, and 7 days, where 0.1, 0.3, 0.5, and 0.7 mg mL^−1^ represent the SF-NF@VSFMP sustained-release system loaded with 0.1, 0.3, 0.5, and 0.7 VSFMPs, respectively.

VANCO can effectively inhibit *Staphylococcus aureus* and has been used in the treatment of various bacterial infections and chronic osteomyelitis (LEW and WALDVOGEL, 2004). However, our previous studies found that VANCO inhibited the activity of MC3T3 during prophase of cell proliferation (SUN et al., 2022). So, the drug concentration should be fully considered in actual use to avoid its side effects as much as possible while treating chronic osteomyelitis (FILIPPONE et al., 2017; WANG et al., 2017). Although SF has good biocompatibility, to ensure that the SF-NF@VSFMPs retain good biocompatibility, they were tested on MC3T3 cells. Considering that SF-NF@VSFMPs have good antibacterial effects, to verify their biocompatibility, the same four experimental parameters were used. As shown in Figure 5, cells on the SF-NF@VSFMPs showed growth after 1, 4, and 7 days of cell culture, and no cytotoxicity was observed due to the increase in VANCO concentration, indicating that each group of SF-NF@VSFMPs had good biocompatibility and the toxicity of loaded VANCO could be ignored. The CCK-8 quantitative analysis results (Figure 4D) showed that the number of cells attached to the hydrogel increased steadily. Some research results show that when the dosage of VANCO exceeds 0.02 mg mL^−1^ or 4 g d^−1^, it may lead to nephrotoxicity (ELYASI et al., 2012). According to our results, the drug loading rate of SFMPs after adsorption in 0.7 mg mL^−1^ VANCO solution is 13.20%. So, the VANCO concentration in the VSFMPs was 0.07 mg mL^−1^. Combined with the sustained-release data of the drug, it could be observed that the concentration of SF-NF@VSFMP sustained-release VANCO within 3 days was not more than 0.007 mg mL^−1^, which is far less than the 0.02 mg mL^−1^ that may cause nephrotoxicity. Meanwhile, Michael et al. showed that many adults can have an adequate VANCO plasma concentration–time curve with a trough concentration of <15 mg L^−1^ (NEELY et al., 2014). Therefore, the release system of VANCO has no biological toxicity, providing an experimental basis for animal experiments *in vivo*.

### 3.6 *In vivo* study of SF-NF@VSFMPs

**FIGURE 6:**
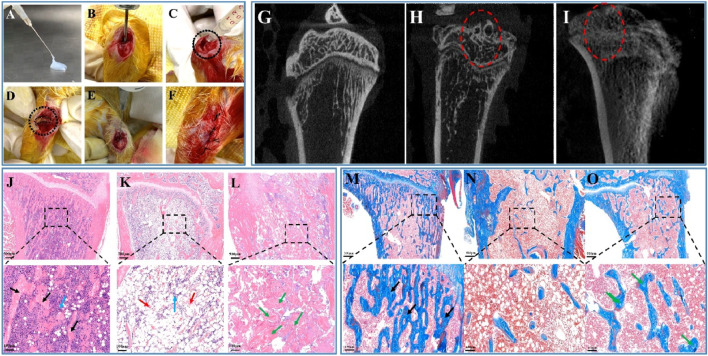
*In vivo* biocompatibility of the SF-NF@VSFMP sustained-release system. Treatment images of chronic osteomyelitis with the SF-NF@VSFMP sustained-release system **(A–F)**, where **(A)** is SF-NF@VSFMP sustained-release system loaded with 0.7 VSFMPs. **(B)** Exposed marrow cavity. **(C)** *Staphylococcus aureus* was injected into the marrow cavity to form a model of chronic osteomyelitis. **(D)** Pus from chronic osteomyelitis wound. **(E)** SF-NF@VSFMP sustained-release system was injected into the area of chronic osteomyelitis. **(F)** Sutured wound. Micro-CT images of the tibia **(G–I)**, where **(G)** is the healthy tibia; **(H)** tibia after the treatment with the injectable SF-NF hydrogel without VSFMPs; **(I)** tibia after the treatment with the SF-NF@VSFMP sustained-release system. Histological examination for tibia **(J–O),** where **(J, M)** healthy animal tibia; **(K, N)** tibia after the treatment of the injectable SF–NF hydrogel without VSFMPs. **(L, O)** Tibia after the treatment of the SF-NF@VSFMP sustained-release system. Note: osteocytes (blue arrow), trabecular bone (black arrow), necrotic tissue (red arrow), and regenerated tissue (green arrow).

The rat model of chronic osteomyelitis and its treatment process are shown in Figures 6A–F. A 1.5 cm incision was made longitudinally at the proximal end of the right tibia to separate the surrounding tissue and expose the tibial plateau. The puncture needle was used in the middle of the tibial plateau to expand the opening and expose the bone marrow cavity (Figure 6B). A total of 50 μL of 1 × 10^7^ CFU mL^−1^ *Staphylococcus aureus* was injected into the opening of the bone marrow cavity, as shown in Figure 6C, and sutured. After feeding for 2 weeks, the wound was opened again to show the chronic osteomyelitis formed in the rat model, as shown in Figure 6D, in which the dotted line is the pus formed by chronic osteomyelitis. According to the sustained release, antibacterial and biocompatibility experiments, 0.7 VSFMP-loaded SF-NF@VSFMPs can be used for the long-term sustained release of VANCO for 25 days, with good biocompatibility and the best *Staphylococcus aureus* inhibition effect, and were therefore selected for this treatment. Figures 6E, F show an image of SF-NF@VSFMPs filling the affected area and the suture, respectively.

In evaluating the treatment and bone repair ability of SF-NF@VSFMPs for chronic osteomyelitis, micro-CT tomography was used to analyze the recovery effect of the affected area and the bone defect repair situation 3 weeks after the operation. Figure 6G shows the healthy tibia of rats. The bone trabeculae in the healthy tibia tissue were evenly distributed and regular. Figure 6H shows the tibial tomography images of rats with chronic osteomyelitis treated with injectable SF–NF hydrogel. There is clear bone trabecula destruction and bone defects in the bone tissue, as well as tissue destruction of the upper tibial segment and cortical blur, indicating that chronic osteomyelitis has caused certain damage to the tibial tissue. After 3 weeks of implantation for SF-NF@VSFMPs (Figure 6I), although the distribution of internal tibial trabecular bone and cortex was not as uniform and clear as that of healthy tibial tissue, there was still a clear trend of bone repair.

The tibia was further analyzed by histological staining. As shown in Figures 6J–O, the internal bone trabeculae of the healthy tibia (Figures 6J, M) were evenly distributed and filled with a large number of healthy bone tissue cells. When *Staphylococcus aureus* was infected and not treated with SF-NF@VSFMPs (Figures 6K, N), there was clear bone trabecular loss, bone tissue necrosis, and cell apoptosis in the bone tissue, indicating serious inflammation. When SF-NF@VSFMPs were implanted into the affected area and treated for 3 weeks (Figures 6L, O), the inflammation was significantly reduced and accompanied by a large amount of new bone-like tissue. Masson staining results also confirmed that SF-NF@VSFMPs had a good therapeutic effect.

## 4 Conclusion

In this study, SFMPs were used as a template to successfully load VANCO to constitute VSFMPs. In order to treat chronic osteomyelitis more effectively, VSFMPs were loaded in an injectable SF–NF hydrogel to form a SF-NF@VSFMP injectable hydrogel, and the therapeutic effect of VSFMPs on chronic osteomyelitis was verified by biological evaluation. The results showed that VANCO was successfully loaded onto SFMPs by physical adsorption. With the increased VANCO concentration, the loading rate of VSFMPs showed an enhanced trend. At the same time, the loaded VANCO did not change the original secondary structure of SFMPs, and VANCO existed on the surface of SFMPs as a crystal structure. The SF-NF@VSFMP sustained-release system could effectively prolong the release period of VANCO to achieve long-term sustained release and had a significant inhibitory effect on *Staphylococcus aureus*, showing a mild and good proliferation effect on MC3T3 cells. Meanwhile, SF-NF@VSFMPs had a good therapeutic effect on chronic osteomyelitis. In summary, this sustained-release system of VANCO will be a potential choice for the treatment of chronic osteomyelitis and bone defects.

## Data Availability

The raw data supporting the conclusion of this article will be made available by the authors, without undue reservation.
